# “Bezoar Egg”—A Rare Cause of Small Bowel Obstruction

**DOI:** 10.3390/diagnostics14040360

**Published:** 2024-02-07

**Authors:** Stefan Milosevic, Jelena Djokic Kovac, Ljubica Lazic, Milica Mitrovic, Katarina Stosic, Dragan Basaric, Boris Tadic, Stefan Stojkovic, Slobodan Rasic, Nenad Ivanovic, Ognjan Skrobic

**Affiliations:** 1 Center for Radiology and Magnetic Resonance Imaging, University Clinical Centre of Serbia, Pasterova No. 2, 11000 Belgrade, Serbia; milosevic.stefan92@gmail.com (S.M.); jelena.kovac@med.bg.ac.rs (J.D.K.); ljbc.lazic@gmail.com (L.L.); katestosic@gmail.com (K.S.); 2 Department for Radiology, Faculty of Medicine, University of Belgrade, Dr Subotica No. 8, 11000 Belgrade, Serbia; 3 Department for HBP Surgery, Clinic for Digestive Surgery, University Clinical Centre of Serbia, Koste Todorovica Street, No. 6, 11000 Belgrade, Serbia; dr.gale@mts.rs (D.B.); tadicboris@yahoo.com (B.T.); 4 Department for Surgery, Faculty of Medicine, University of Belgrade, Dr Subotica No. 8, 11000 Belgrade, Serbia; nekic85@gmail.com (N.I.); skrobico@gmail.com (O.S.); 5 Clinic for Gastroenterology and Hepatology, University Clinical Centre of Serbia, Koste Todorovica Street, No. 2, 11000 Belgrade, Serbia; stefanstojkovic@ymail.com; 6 Department of Stomach and Esophageal Surgery, Clinic for Digestive Surgery, University Clinical Centre of Serbia, Koste Todorovica Street No. 6, 11000 Belgrade, Serbia; bokirasic92@gmail.com

**Keywords:** bezoar, small bowel obstruction, intestinal stricture, radiation enteropathy, intestinal stricture, ileus

## Abstract

Small bowel obstruction is a frequent medical condition with various causes, the most common being postoperative adhesions, volvulus, intussusception, hernias, and tumors. A bezoar-induced blockage of the small intestine is a rare condition that accounts for approximately 4% of all small bowel obstruction cases. Herein, we present the case report of a 71-year-old patient with diffuse abdominal pain caused by a small bowel obstruction due to a calcified bezoar (bezoar egg) resulting from a post-radiation intestinal stricture. The patient underwent a small bowel excision with the extraction of the bezoar, after which a full recovery was made.

A 71-year-old female patient was admitted to our department with diffuse abdominal pain and subocclusive symptoms in the form of bloating, an absence of stool, nausea, and occasional vomiting. Over the past two years, she has been treated several times in the emergency department for the abovementioned symptoms. In her medical history, the patient reports an appendectomy three years ago and radiotherapy for cervical cancer 25 years ago. Physical examination revealed diffuse moderate abdominal pain. The abdomen felt tender on touch. The laboratory tests were within the reference values for the patient’s age, her leukocyte count was 9.2 × 10^9^/L, erythrocyte count was 4.9 × 10^12^, hemoglobin concentration 142 g/L, alpha-amylase 49 U/L, pancreatic-amylase 28 U/L, aspartate aminotransferase 34 U/L, alanine aminotransferase 20 U/L, alkaline phosphatase 66 U/L, and gamma-glutamyl transferase 14 U/L, except for the C- reactive protein which was slightly elevated at 10.5 mg/L.

Abdominal X-ray showed centrally located, dilated small bowel loops up to 4 cm in diameter, with gas–fluid levels and no signs of pneumoperitoneum (Figure 1A). Abdominal ultrasonography confirmed the presence of dilated small bowel loops filled with a dense liquid and showing signs of antiperistalsis. Interintestinally, a laminar layer of free fluid was seen. Deep in the pelvis, an oval calcified formation approximately 4.5 cm in diameter was seen blocking the small bowel lumen, with a subsequent collapse of the distal bowel loops (Figure 1B).

A computed tomography (CT) scan also confirmed the presence of several dilated loops of the small bowel up to 3.5 cm in diameter, most of which were located in the pelvis. An oval, clearly demarcated mass with centrally placed gas inclusions was visualized in the lumen of one of the ileal loops (Figure 2A). A ring-shaped, circumferential thickening of the ileum wall was seen directly adjacent to the formed mass (Figure 2B). There were no signs of perforation or hemorrhage on the CT.

To rule out the possibility of a polipoid tumor originating from the small bowel wall, a magnetic resonance imaging (MRI) examination was performed.

The MRI showed more clearly the layered structure of the described mass due to its better tissue resolution. The low signal intensity at the periphery of the lesion itself corresponded to the calcified component. There was no diffusion restriction in the diffusion-weighted imaging sequence or post-contrast opacification of the lesion. Beneath this clearly formed, calcified bezoar, in the form of a “bezoar egg”, a circumferential narrowing of the ileum segment was observed, which showed the imaging features of a benign fibrous stenosis of the lumen in all cross-sectional examinations performed (Figure 3A,B). Regarding previous radiotherapy, this could be a consequence of the therapy and practically the cause of the bezoar forming with subocclusive symptoms.

A resection of the small bowel was performed over a length of about 10 cm with the formation of an entero-entero anastomosis. An oval solid lesion approximately 4.5 cm in diameter was retrieved from the small bowel lumen, primarily corresponding to a bezoar caused by the chronic deposition of intestinal contents (Figure 4A,B). Near the ileal loop from which the bezoar was extracted, a circular zone of bowel stricture was observed and resected (Figure 4C) (watch Video S1 in the Supplementary Materials). Pathohistological analysis confirmed the presence of chronic ulcero-fibrous stenosis of the bowel wall, which caused the formation of the bezoar without elements of cellular atypia, dysplasia, or neoplastic infiltration. The patient tolerated the operation well and made a full recovery.

Bezoars are solid masses formed by the fusion of indigestible substances in the gastrointestinal tract [1]. They can be categorized into different groups based on the sources of their constituents, including phytobezoars, trichobezoars, drug bezoars, and lactic acid bacteria bezoars. [1,2]. A calcified bezoar is very rare, most likely due to the prolonged time it takes to form. They are typically found in the stomach and can migrate through the pylorus into the small intestine, leading to small bowel obstruction [3].

In rare cases, bezoars can originate in the small intestine and are often associated with common conditions such as diverticula, tumor formation, or strictures [1]. Some common causes of stricture of the small bowel lumen are Crohn’s disease, ischemic injury, certain infections such as tuberculosis, tumors, and radiation therapy [4]. Our patient had a short, annular, segmental thickening of the intestinal wall with a calcified bezoar immediately proximal to it. It is very likely that the stenotic segment formed as a result of radiotherapy, with subsequent bezoar formation and intermittent subocclusions.

It is difficult to distinguish the clinical symptoms of bezoar-induced small bowel obstruction (BI-SBO) from those caused by other factors leading to intestinal obstruction [5,6]. The most common manifestations are complete mechanical intestinal obstruction, leading to symptoms such as abdominal pain, bloating, nausea, and vomiting [2,6]. Relying solely on clinical symptoms can lead to a delay in BI-SBO diagnosis and surgical treatment, which can increase morbidity and mortality [3,7]. In cases where BI-SBO patients have a history of gastrointestinal surgery or laparotomy, clinicians may initially assume an adhesive intestinal obstruction and perform conservative treatments, which in our case led to repeated hospitalizations of the patient. Therefore, it is crucial to choose appropriate imaging studies to diagnose BI-SBO in its early stages so that immediate clinical intervention is possible [3]. Early surgical intervention is crucial and feasible for the effective treatment of BI-SBO, since delayed treatment significantly increases the risk of complications and mortality [8]. Therefore, timely and appropriate medical treatment is crucial in the management of this condition.

The diagnosis of small bowel obstruction is highly dependent on imaging studies, especially CT. With a diagnostic sensitivity of 73% to 95% and an accuracy of 83%, CT is used not only to accurately determine the causes, location, and degree of obstruction, but also to assess the presence of intestinal ischemia [9,10]. Mottled gas density is a typical feature of bezoars on CT that can be used to differentiate them from stool [11,12]. In some rare cases, a bezoar may appear as a soft tissue mass without gas that resembles an intraluminal tumor. In these cases, MRI, with its excellent soft tissue contrast, can accurately visualize the bezoar and precisely delineate it from the intestinal wall [13]. Since, in our case, the bezoar was calcified, the main differential diagnosis was gallstone ileus. But with no cholecysto-enteric fistula and the absence of gas in the biliary tract this diagnosis was excluded. Also, possible alternative diagnoses included a calcified polyp or calcified leiomyoma of the small bowel and an ingested foreign body, which were also excluded based on imaging features (Table 1).

BI-SBO is a rare diagnosis that poses a challenge both in terms of its diagnosis and management. It should be considered in patients with an increased risk of gastrointestinal bezoar formation, e.g., patients with a history of gastric surgery, poor dental hygiene, and increased fiber consumption [14]. In high-risk patients and patients with SBO, an early CT scan is recommended, regardless of previous abdominal surgical interventions. This approach aims to minimize unwarranted delays before performing the necessary surgical intervention.

## Figures and Tables

**Figure 1:**
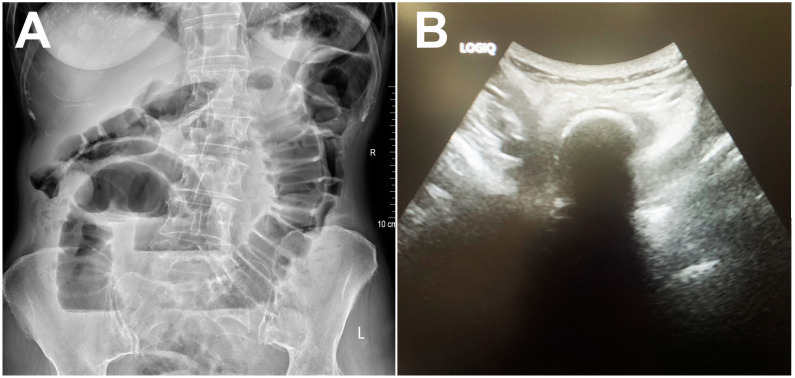
Abdominal X-ray showing dilated small bowel loops with gas–fluid levels (**A**). Grayscale abdominal ultrasound showing hyperechoic oval structure with posterior acoustic shadowing within the bowel lumen (**B**).

**Figure 2:**
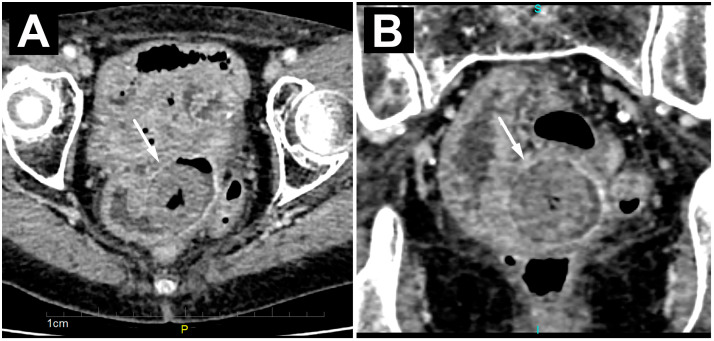
Axial (**A**) and coronal (**B**) CT images of an enclaved bezoar (white arrow) level with a ring-like, circumferential thickening of the ileum wall, with consequent proximal dilatation of the small bowel loops.

**Figure 3:**
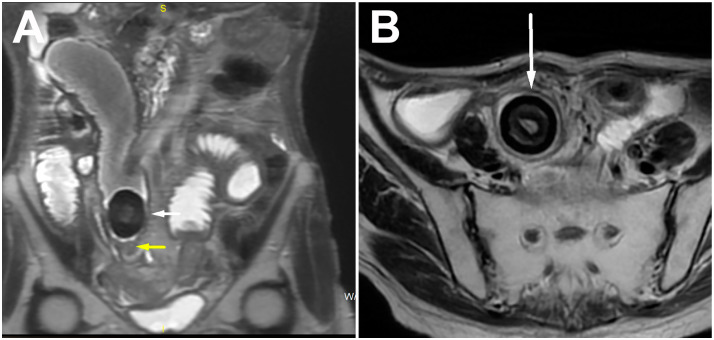
Coronal (**A**) and axial (**B**) T2w images showing ring-like stenosis of ileum (yellow arrow) and layered structure of bezoar (white arrow), with very low signal intensity on the periphery representing calcified component.

**Figure 4:**
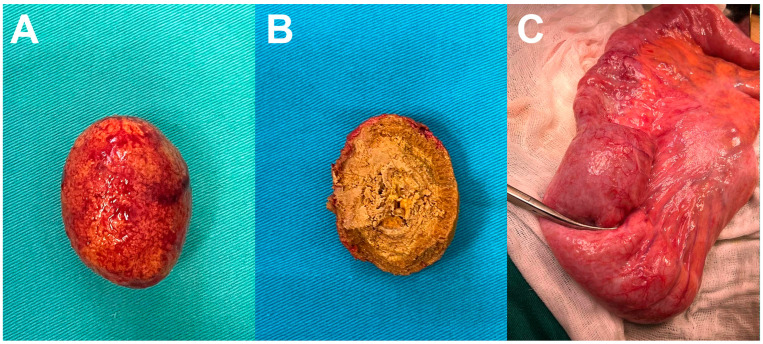
Macroscopic appearance of “bezoar egg” (**A**) and its layered structure (**B**). Circumferential zone of intestinal stricture with intraluminal “bezoar egg” proximal (**C**).

**Table 1:** Main imaging features for the differential diagnosis of calcified bezoars [1,11,13].

Intraluminal Causes of SBO	Ultrasound	CT	MR
Calcified bezoar (“Bezoar egg”)	Hyperechoic oval structure with posterior acoustic shadow within the bowel lumen	Oval, clearly demarcated mass with centrally placed gas	T1w and T2w hipointense oval mass with layered structure
Non-calcified bezoar (trichobezoar, phytobezoar, lactobezoar)	Arc-like surfaced intraluminal mass with posterior acoustic shadow	Oval or tubular masses with clear boundaries located inside the intestinal lumen, with mottled gas density	Oval mass with low T2w signal and a low to intermediate T1w signal intensity
Gallstone ileus	Hyperechoic oval structure with posterior acoustic shadow	Oval calcified or isodense intraluminal mass, presence of pneumobilia and cholecysto-enteric fistula	Oval mass with a homogenously low signal intensity on T1w and T2w
Primary small bowel neoplasm (calcified polyp, calcified leiomyoma)	Oval soft tissue mass with posterior acoustic shadow	Intraluminal, completely or partially calcified mass that arises from the bowel wall	Low T1w signal intensity; heterogeneous to high T2w signal intensity with central area of enhancement representing a central vascular stalk and focal areas of signal void representing calcifications
Ingested foreign body	Variable shape and structure (depending on the type of ingested foreign body) with posterior acoustic shadow	High density, intraluminal variable shape and structure	Uniformly low signal intensity on T1w and T2w sequences

## Data Availability

The datasets used and analyzed in this paper are available from the corresponding author on reasonable request.

## Supplementary Materials

The following supporting information can be downloaded at https://www.mdpi.com/article/10.3390/diagnostics14040360/s1, Video S1: intestinal stricture and “bezoar egg” proximal in the bowel lumen.
